# Certain Points in Diphtheria

**Published:** 1877-09

**Authors:** F. C. Curtis

**Affiliations:** Albany


BUFFALO

Medical and Surgical Journal.

VOL. XVII. SEPTEMBER, 1877. No. 2.

### Original Communications.

:o:

ART. I.—*Certain points in Diphtheria* By F. C. CURTIS, M. D., Albany.

Diphtheria has attracted a good deal of attention in the medical journals and pamphlets for some time back, due, no doubt to more or less prevalence of the disease in certain localities. In New York especially, it is known that a long continued epidemic is prevailing, and from there as from other places, a great diversity of views regarding this disease have been made public through the medical press, or in society reports. In Albany there has been no extensive epidemic for some years, although sporadic cases have been constantly occurring, which, during the past winter have been more numerous; but not to be dignified with the term of epidemic, for the recently issued report of the city registrar for the year ending April 30th, last shows but 29 deaths from this disease out of a total death rate of 1138. Still we have had some experience with the disease.

I wish to speak of a few points only connected with Diphtheria. Etiology is an uncertain subject, yet exactness is important here. It is a question of practical interest how much vegetable parasites, or bachteria have to do in producing and keeping up the disease. The bacterian theory seems to be going out of fashion, but the earnest endorsement of it by Oertel and others, renders it still worthy of respectful consideration. In the lengthy article in Ziemssen's Cyclopædia, by Oertel, on Diphtheria, (now written, to be sure, some time ago,) he describes the various forms of these lowly organized vegetations found on the mucous membranes, one of which, the spherical bacterium or micro-coccus, he considers characteristic of diphtheria. The other forms, all of them may be found present in any simple pharyngitis, and especially such as is attended with the formation of membrane, as the croupus, but the micro-coccus never is seen, provided diphtheria does not set in. This spore possesses this peculiarity—that existing along with other fungi, although in ever so small a quantity, it rapidly increases, grows vigorously and soon overgrows and crowds out others and destroys them. In short this extensive observer and experimenter tells us *that micro-cocci are inseparable from the diphtheristic process, and without micro-cocci there can be no diphtheria.* Taking this as a primary proposition, this existence and growth of a fungus on the faucial membrane, it is believed that the disease becomes constitutional by the absorption of these fungi—or at any rate of some septic matter—from this primary location into the system. Oertel says that the bacteria are absorbed, for he finds them in the blood vessels, filling them and mechanically damming them up; muscular fibres are crowded with them; the kidneys contain them in immense numbers, heaped up in the tubules and malpighian corpusles. How they produce their sceptic effect, he leaves undetermined. Others who allow that the disease becomes constitutional by absorption from the fauces hold that it is a virus peculiar to the disease that is absorbed; still others think that it is not a specific virus, but a septic poison absorbed from the inflamed surface which is not different from the poison of ordinary septicæmia. Whether this bacterian theory is correct or not, I will not stop to argue, as it is not necessarily to my purpose. It is an extremely materialistic view of the pathology of diphtheria, peculiarly German, and has many facts opposed to it, some of which Dr. J. Lewis Smith, in one of the most satisfactory articles I have read, specifies: the micro-cocci are found in localities where diphtheria has not occurred; they are even found on the gums of healthy persons; they do not affect the lungs, although they must reach them by inspiration as readily as the fauces. But whether the *muteries morbi* is the micro-coccus or something not yet determined, I believe the ground well taken that in the majority of cases it has its existance upon the fauces, and that if it remains there it is absorbed into the system, and that its tendency is when thus absorbed to produce a fatal issue. I arrive at this conclusion from clinical deductions, having observed no case of recovery in young subjects, when the disease was well marked in which a treatment on "general principles," or on the expectant plan was pursued, and on the other hand about all have recovered who received local specific treatment from the start, and it is a point of very great importance in the study and treatment of this virulent disease as to whether or not the virus can be destroyed *in situ*, on the surface of the body as it were, before it affects vital structures. It has been taught, and still is, that the main point of difference between croup and diphtheria is that the one is always local, and the other always constitutional. Undoubtedly this statement is true, but is diphtheria essentially a constitutional disease from its inception, even though attended as it usually is from the first with headache and fibrile action. May not these general disturbances be dependent upon the local pathological process? For to repeat, good results have only followed, so far as my observation has gone, where a remedy has been exhibited, which, if not applied directly to the throat, was given with a view to its coming and remaining in contact with the affected surfaces for a time. I think that the reported success of various remedies given internally has been due to a local effect; there is hardly one of them mentioned as efficatious in the journals that we may not conceive as having a decided local influence, as for instance the hyposulphite of soda which some regard as a specific. To be sure there is a possibility of bracing up the system to so high a pitch that the effect of a poison may be resisted.

For snake bites the popular specific is whiskey in large quantities —a constitutional treatment for a poison received locally, but so rapidly absorbed as to get speedily beyond local reach. But may it not be that in much the same way a curative effect was produced by quinine and large doses of alcoholics, which Dr. Alonzo Clark used to instruct us to rely on, the efficacy of the latter having been, he says, accidently found out through a nurse giving at a short time, nearly a bottle of wine to a child sick with diphtheria, improvement following. The view of Dr. Delafield of New York, that, as I understand it, this disease is a compound one, like epidemic influenza, cerebro-spinal fever and a few others made up in its pathology of an essential fever and a local inflammation, may offer a way to reconcile the phenomena of the disease better than the various theories that would make it either strictly local or constitutional primarily. A point that bears against its being one of the acute infectious diseases, purely constitutional, and one which I do not remember to have seen enforced, is its frequent co-existence with other acute infectious diseases. It is well known to frequently graft itself upon measels and scarlatina. And in hospitals where hospital gangrene or puerperal fever is prevailing it is apt to come in as an unfortunate complication of recent operations. With scarlatina there seems to be sometimes a mutual modification so that it is difficult to say which is the primary, essential disease. But whatever view we may take of the nature of the disease, my belief is that it is a poison to the system, whose natural tendency is to cause death, and that ignoring the local manifestation usually ensures this result, unless the virulence of the disease or amount of the poison is small, in other words the case a mild one, or on the other hand the subject strong to resist it, as we see in the case of adults.

Bearing on these points as well as upon the anti-hygienic conditions favoring its development, I will relate the cure of a single family. About the middle of last December I went to see the child of an employee at the Rural Cemetery, living in the burying ground of the jews on the elevated ground just back of the cemetery. He had just lost a child with diphtheria, dying the day before I was called to see this one. Of the nature of the malady there was no question. My case was a child five years of age; she had been taken sick the day before. There was deposit on both tonsils about the size of a little finger nail, a thin pellicle, having the crescentic outline suggestive of parasitic development, and looking as if just under the superficial layer of the mucous membrane. This interstitial deposit is insisted on as characteristic by Virchow. Such an appearance as this showed thus early is never presented by any other deposit on the fauces. The cervical glands were a little swollen; the child had an apathetic look and was disinclined to make any muscular effort. There were four other children in the family, two older,—10 or 12 years old, and two younger—about 4 and 1½ years old. All of them had moderately inflamed throats and enlarged cervical glands, but no deposit, and they were all around and well. I directed the treatment recommended by Dr. J. Lewis Smith* to be at once instituted. For a local application this mixture,—℞: *Acid. Carbol.* gtt. x; *Liq. ferri subsulphat.* ℥iii *Glycerine* ℥i, to be applied every two hours with a moderately large soft camel-hair pencil, which was to be washed out thoroughly after each use, and left in a glass of frequently renewed water. Nothing was to be given to drink for 15 or 20 minutes after the application. I also gave every four hours a small teaspoonfull of ℞: Potas. chlor. ʒiss. Tr. ferri chlor. ʒi. Syr. Simplic. ℥ii. This solution smells distinctly of chlorine, which is the most perfect destroyer of disease germs that we possess. No drinks were to be given for a few minutes after each dose of this, I gave her a grain of quinine every four hours, and the same to the other children. Next day the membrane was thick, it was dark brown from the iron, it lays now on top of the mucous membrane distinctly. It had extended all over the tonsils and up into the posterior nares as far as could be seen. There was a vast amount of nasal discharge, thin pus. The child was docile, and the remidies had been faithfully applied. I directed them to be continued. Next day the edge of the white membrane could be distinctly seen coming down into both nostrils. The pulse was 110 and thready, and the patient was very weak. I took a small glass syringe about the size of the ring finger, intended perhaps for a vaginal syringe, filled all the holes but the center one with wax, and directed a third of a teaspoonful of the mixture of carbolic acid and iron to be put into a small quantity of warm water, and injected into both

* Diseases of Infancy and Childhood, 3d Ed., p. 242.

nostrils once in three hours. She was disposed to gargle, and I gave for this a solution of chlorate of potash containing carbolic acid. Whiskey was prescribed, but was not taken largely for the child, otherwise very tractable, could not be persuaded to take much of this. For two days now there was little change in the condition of the throat, the thick leathery membrane continuing adherent, and no attempt was made to remove it mechanically. Then it began to come away freely on the brush, and in a little while was entirely off. The application diluted was continued at large intervals, but produced much pain. The child mended rapidly from grave symptoms, asthenia continuing for a long time, as well as the return of fluids through the nose on the attempt to swallow them. She also had strabismus which after a time disappeared. My last prescription for her was citrate of iron and strychnia. A few days after this child was taken the next younger began to complain, and her throat was found by her parents to present small spots of deposit, quite such as seen on the other, only smaller. They immediately made applications of the throat mixture of his sister, using a separate swab, and the deposit developed no further, disappearing in a couple of days, and she had very little after malaise. I only saw this deposit after it was stained with iron, and cannot speak certainly as to its character. I had given directions that upon the least complaint, if any deposit showed, the solution should be applied at once. About four weeks after the appearance of the disease in the family, the babe was taken in the same way. I only saw this case once, and found that they had the idential tseatment of the first case. The deposit when seen was thick and circumscribed, the child showing but little constitutional disturbance. The treatment was kept up, and she made a good recovery. The father was taken subsequently in April, and employed the same treatment as the others. He recovered easily.

What I wish to illustrate by these cases is the fact, that the treatment was almost purely antizymotic and local. I think I have said enough to show the nature and severity of the disease. What the treatment was in the case of the child that died, I do not know, but believe it was simply sustaining. Others have used what might be called an antizymotic treatment, exhibited internally. Dr. Burton of Fultonville tells me that he has habitually used, and with remarkable success, a solution of hyposulphite of soda. We may assume that free sulphurous acid forms in this solution, and that this is the destructive agent of the disease germs. Such administration of medicines I should prefer to make in glycerine as a vehicle, to help its fixing on the throat. Chlorine is a better agent for destroying disease germs than sulphurous acid, being used for fumigation of houses infected with small pox, in preference to sulphur fumes, and we should look for a good effect from it, though it has the disadvantage of being difficult to administer, except in minute quantity. If, on the other hand, however, it is the azone formed at the moment of the generation of chlorine gas that is the efficient agent, we should look for no effect except from a solution constantly generating chlorine. As to the value of carbolic acid as an antizymotic it is not necessary for me to speak. It is well known that the mineral salts generally have a similar action, and the large doses of muriated tincture of iron recommended by some, or the iodo-bromide of calcium comp, etc., may all have had their effect, partially at least, in such a way. But theories of treatment should all be thrown aside and empyricism made the test, and while we may not draw conclusions from a few cases, the experience of all help. Theory may avail to reconcile apparently conflicting experiences to each other. But with all I must say that I do not believe we treat diphtheria to our best ability, unless we add quinine and whiskey to whatever antizymotic is employed. The vital forces must be supplemented with all our power in this exhausting disease, especially when cases are not seen at their very inception.

An interesting point connected with the cases in the family I have spoken of, is how much their anti-hygienic surroundings had to do with the production of the disease. They lived in a grave yard on the back of a long rolling ridge that is well drained, not far away is an abrupt ravine through which was a small stream which rises outside of the cemetery, and runs through it. The wells of this family and of another near by, also in the cemetery, are thirty or forty feet deep, about the level of the creek bed. The water of both is bad and that of the latter smells strongly of sulphuretted hydrogen, which has however, established its reputation as possessing medicinal properties, and visitors to the cemetery frequently carry it away. Dr. Willis G. Tucker had the kindness to examine for us, specimens from these wells and found them unfit for use, the one used by the family spoken of being especially bad water, having an offensive odor and containing a large quantity of decomposable organic matter, ammoniacal salts, nitrites and chlorides, being also very hard and holding carbonate and sulphate of lime and magnesia. The nearest graves were 25 feet away, the remains having lain there two years and upwards. Large quantities of petrefactions have been found in old graves when dug open.

The creek water was not pure, but was better. This family has used their well water for years and have been uniformly healthy, no zymotic disease affecting them. The only difference in circumstances is that the water is unusually low in the wells. Throughout the cemetery grounds there are numerous springs and streamlets all flowing off freely, as they have a rapid fall. The water from them is used freely and constantly by the laborers. Diphtheria has not existed to my knowledge on the rising ground back of the cemetery, excepting in the family spoken of, but at the lower front of the cemetery,and alongthe Troy road a good many cases have been occurring. No conditions favoring its development are to be found there, other than those mentioned, except that the ground is low and flat towards the river. Individual efforts to study up the etiology of disease as well as the prevention of endemics and epidemics cannot avail a great deal. It is most earnestly to be hoped that the time is not far distant when we shall have a state Board of Health and efficient local Boards, to whom, among other things, it shall be required that every case of zymotic disease must be reported. This combination of effort will then render efficient the work of individuals, and by means of it causes for endemics may be discovered and removed. This prevention is earnestly to be desired at least in connection with so virulent a disease as the one I have been considering.

